# Recent advances and future directions in banana molecular biology and breeding

**DOI:** 10.1186/s43897-024-00122-2

**Published:** 2024-12-02

**Authors:** Chunzhen Cheng, Shuofan Wu, Guiming Deng, Ou Sheng, Ganjun Yi, Qiaosong Yang

**Affiliations:** 1 Institute of Fruit Tree Research, Key Laboratory of South Subtropical Fruit Biology and Genetic Resource Utilization, Ministry of Agriculture and Rural Affairs, Guangdong Provincial Key Laboratory of Science and Technology Research On Fruit Tree, Guangdong Academy of Agricultural Sciences, Guangdong, 510640 China; 2 College of Horticulture, Shanxi Agricultural University, Jinzhong, 030801 China; 3 Maoming Branch, Guangdong Laboratory for Lingnan Modern Agriculture, Guangzhou, Guangdong, 510640 China

**Keywords:** Banana (*Musa* spp.), Molecular biology, Fruit ripening biology, Stress resistance, Breeding

## Abstract

Since publication of a draft genome of the doubled-haploid ‘Pahang’ banana (*Musa acuminata*, DH-Pahang), a new era for banana biology research has begun. With the release of genomic data from some important *Musa* species and subspecies and the continuous development of molecular biology techniques, significant progress has been made. Here, we summarize the achievements and advances in the banana molecular biology and breeding over the past decade covering origin and domestication, fruit biology, stress biology, and breeding aspects, and highlight their challenges and future perspectives. This review is intended to provide researchers with the latest information on the complex genetic background and evolutionary relationship of bananas, the biology of fruit ripening, and multi-omics-based stress biology research. We especially focus on recent advances in the molecular breeding of bananas, offering an informative research direction and providing valuable technical references for future research in the field.

## Introduction

Bananas (*Musa* spp.), including plantain, are one of the world’s essential fruits and food crops. They are cultivated in tropical and subtropical regions more than 130 countries. Based on FAOSTAT’s recent data, banana annual production is about 179.26 million tons, which occupies over 12.67 million hectares of land globally (FAOSTAT 2022). Given the sterile and polyploidy characteristics of most cultivated bananas and their unjustified ancestors, their origin and domestication receives extensive attentions (Heslop-Harrison and Schwarzacher 2007). Fortunately, the interpretation and publication of the genomes of some *Musa* species and subspecies has significantly clarified and leveraged their enhanced breeding (Huang, et al. 2023; Li, et al. 2024; Xie, et al. 2024).

In recent years, there have been growing challenges of banana production in the international banana industry. These include a short shelf-life, sensitivity to several common abiotic stresses (low temperature, drought, salinity and others) and susceptibility to biotic stresses (Fusarium wilt, bacterial wilt, viruses, nematodes, etc.). Breeding new varieties is one of the most effective ways to address these problems. However, given the limited success of cross breeding, lack of genomic resources for banana cultivars, and insufficient molecular markers, the field in banana breeding has progressed slowly, remaining with large knowledge gaps. Therefore, it is important to understand the complex genetic background and evolutionary relationship of bananas and characterize the high-yield, high-quality, and resistance genes at multiple levels.

Publication of the draft genome of the doubled-haploid ‘Pahang’ banana (*M. acuminata*, DH-Pahang) in 2012 (D’Hont, et al. 2012), has opened a new, fast-developing era for banana research. Significant efforts were made to not only sequence genomes of *Musa* species and subspecies but also combine multi-omics technologies (including transcriptomics, proteomics, metabolomics, epigenomics, etc.), which have provided a comprehensive understanding of the genetic and molecular mechanisms underlying banana biology, paving the way for innovative approaches in banana breeding, disease resistance, and crop improvement. Here, we review the advances in banana molecular biology over the past decade, covering from its origin and domestication to improvement of its fruits and resistance, and breeding alternatives. Additionally, we highlight the challenges and future perspectives of banana research.

## Origin and domestication of bananas

Bananas are large perennial herbs mainly distributed in tropical and subtropical regions of Asia and Oceania, with outliers in Nepal and south part of China to the north, Queensland to the south, Pemba Island to the west, and Samoa to the east (De Langhe, et al. 2009; Xiao, et al. 2024). The northern and southern outliers are considered natural, but the outliers in the west and east are anthropogenic (De Langhe, et al. 2009). Bananas are generally thought to be originated in Southeast Asia and the west Oceania (Wang, et al. 2019b), with New Guinea considered as one of the earliest origin centers of wild bananas (Denham, et al. 2003).

**Fig. 1:**
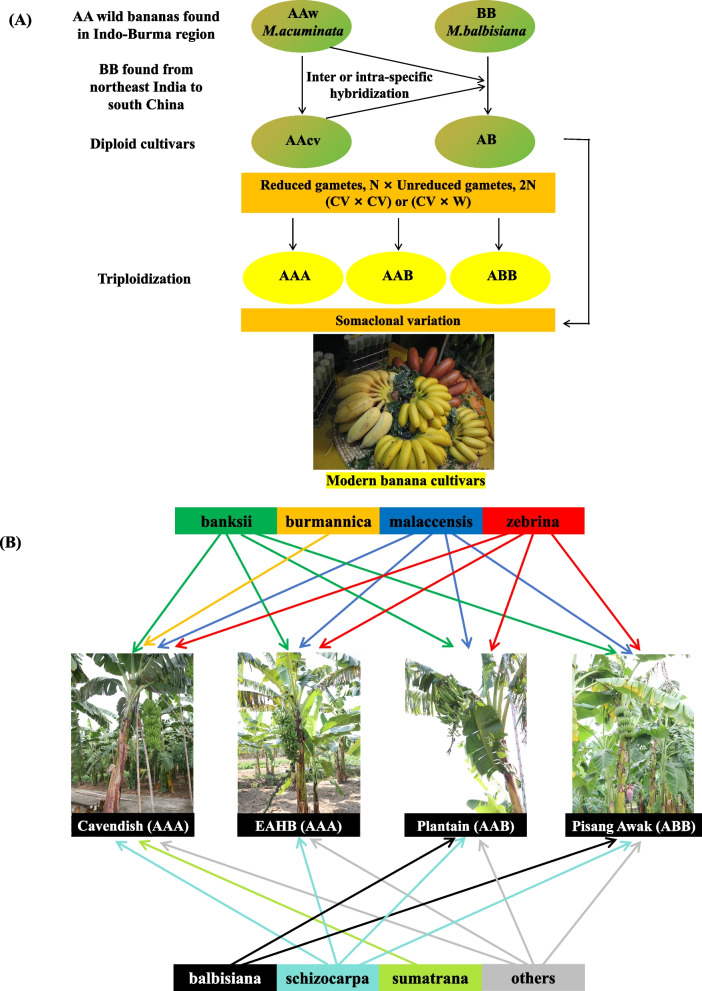
Diagram of the evolution of modern cultivated banana (**A**) and genome ancestry of modern triploid banana cultivars (**B**). banksii: *M. acuminata* ssp. *banksii*; burmannica: *M. acuminata* ssp. *burmannica*; malaccensis: *M. acuminata* ssp. *malaccensis*, zebrina: *M. acuminata* ssp. *zebrina*, balbisiana: *M. balbisiana*; schizocarpa: *M. schizocarpa*; sumatrana: *M. acuminata* ssp. *sumatrana*; Cavendish accession name: Grande Naine; EAHB (east African highland banana) accession name: Intokatoke; Plantain accession name: Mkono Wa Tembo; Pisang Awak accession name: Namwa Khom

*Musa* is divided into two clades: one is comprised of the sections *Ingentimusa*, *Callimusa*, and *Australimusa* (2n = 14, 18, or 20); the other is of the sections *Eumusa* and *Rhodochlamys* (2n = 22) (Häkkinen 2013). According to their genomic compositions, bananas are predominantly categorized into AA, AB, AAA, AAB and ABB genotypes, as well as some BBB, TT, AT, AAAB, AABB, ABBB (Wang, et al. 2019b; Zhou, et al. 2024) and limited AS, AAT and BBT genotypes (Rekha 2016). Accumulated evidence has verified that the majority of the cultivated, seedless triploid bananas have evolved from two wild species, *M. acuminata* (A genome, 2n = 22) and *M. balbisiana* (B genome, 2n = 22), through natural inter/intra-specific hybridization and somaclonal variations (Fig. 1A) (Simmonds and Shepherd 1955; Rekha 2016). Further, it appears that *M. schizocarpa* (S genome, 2n = 22) and some species from the *Australimusa* section (T genome, 2n = 20) have also contributed to the genetic composition of modern banana cultivars (Fig. 1B) (Huang, et al. 2023; Martin, et al. 2023).

Due to its separated spatiotemporal domestication (De Langhe, et al. 2009), polyploidy and mostly vegetative propagation characteristics, plus the intentional and unintentional impact of human, the domestication of bananas is extremely complicated. However, given the diverse ancestral contributions and some missing ancestors, similar botanical traits of some cultivated bananas, lack of some important genetic resources, and limited number of markers (Martin, et al. 2020), the distinguishing of many cultivated bananas is often very difficult. Over the past decade, genomics and some other omics techniques have been successfully applied in exploring the genetic diversities of both wild and cultivated bananas and some other *Musa* species, which have greatly facilitated the clarification of the domestication of cultivated bananas (Martin, et al. 2020; Huang, et al. 2023; Li, et al. 2024). A few *Musa* genomes, including the improved genome of DH-Pahang, those of some wild banana resources and their wild relatives have been assembled and released. These include *M. balbisiana* (Wang, et al. 2019b), *M. itinerans* (Wu, et al. 2016), *M. schizocarpa*, *M. beccarii* (Wang, et al. 2023), *M. textilis* (Zhou, et al. 2024), *M.troglodytarum* (Zhou, et al. 2024), *M. ornata* and *M. velutina* (Xiao, et al. 2024), providing the most abundant genomic datasets and molecular markers for clarifying the domestication of cultivated bananas.

*M. acuminata* is the most important genetic resources for cultivated bananas, contributing to the fruit quality and parthenocarpy of the modern species (Heslop-Harrison and Schwarzacher 2007). It can be divided into multiple subspecies, among which at least five have been identified as contributors to cultivated banana varieties, namely *banksii*, *zebrina*, *malaccensis, burmannica* and *errans* (Perrier, et al. 2011). Through multivariate analysis and SNP clustering, five possible ancestral contributors to cultivated AAA bananas were identified (Fig. 1B) (Martin, et al. 2020; 2023). Phylogenomic analyses of ‘Banksii’ (*M. acuminata* ssp. *banksii*), ‘Maia Oa’ (*M. acuminata* ssp. *zebrina*), and ‘Calcutta 4’ (*M. acuminata* ssp. *burmannica*) suggest that rapid radiation within *M. acuminata* subspecies occurred after its divergence with *M. balbisiana*. Introgression between *M. acuminata* ssp. *malaccensis* and *M. acuminata* ssp. *burmannica* was detected across the genomes (Rouard, et al. 2018). Most dessert bananas belong to the ‘Cavendish’ and ‘Gros Michel’ subgroups. Recently, their chromosome-scale genome assemblies revealed that the three A subgenomes are with *M. acuminata* ssp. *banksii* (endemic to New Guinea), *malaccensis* (originated from the Malay Peninsula) and *zebrina* (found in Java island in Indonesia) as major ancestral contributor (Li, et al. 2024), respectively. This finding supports the geographical distribution of wild *M. acuminata* bananas.

## Advances in banana fruit ripening biology

### Regulation of banana fruit ripening

**Fig. 2:**
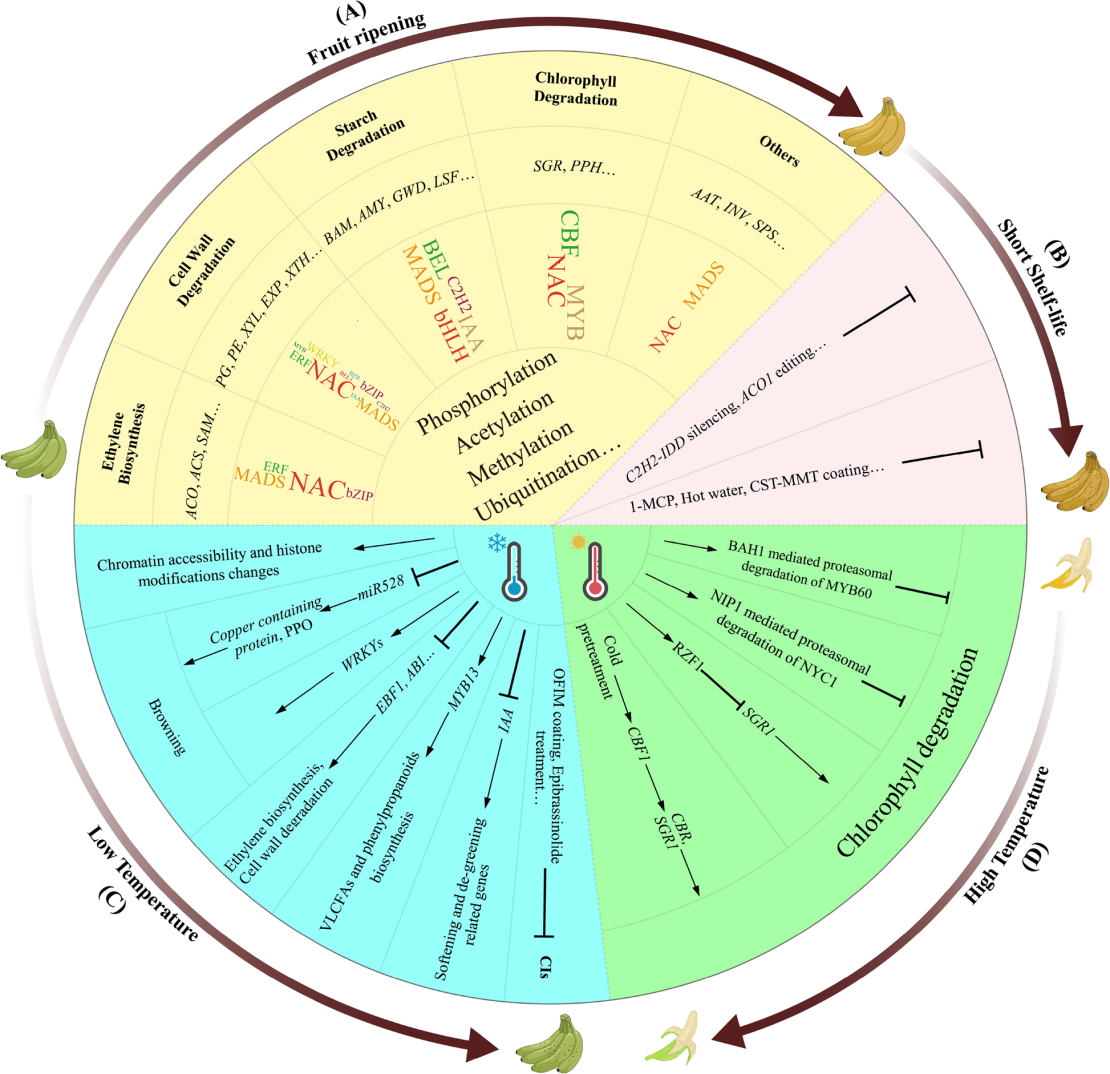
Summary of recent advances in banana fruit biology. **A** Identified genes and other factors influencing fruit ripening; (**B**-**D**): Reported genes and methods used to address industrial issues in banana production. *ACO*, *1-aminocyclopropane-1-carboxylic acid oxidase*; *ACS*, *1-aminocyclopropane-1-carboxylic acid synthase*; *SAM*, *S-Adenosyl-L-methionine synthetase*; *PG*, *polygalacturonase*; *PE*, *pectinesterase*; *XYL*, *β-D-xylosidase*; *EXP*, *expansin*; *XTH*, *xyloglucan endotransglucosylase/hydrolase*; *BAM*, *β-Amylase*; *AMY*, *α-Amylase*; *GWD*, *glucan, water dikinase*; *LSF*, *starch-related phosphatase Like-Sex-Four*; *SGR*, *stay-green*; *PPH*, *pheophytinase*; *AAT*, *alcohol acyltransferase*; *INV*, *invertase*; *SPS*, *sucrose-phosphate synthase*; 1-MCP, 1-methylcyclopropene; CTS-MMT, chitosan-montmorillonite; *EBF1*, *ethylene F-box 1*; *ABI*, *abscisic acid-insensitive-like*; VLCFAs, very-long-chain fatty acids; *PPO*, *polyphenol oxidase*; CI, chilling injury; OFIM, *Opuntia ficus indica* mucilage; BAH1, benzoic acid hypersensitive 1; NYC1, nonyellow coloring 1; NIP1, NYC1-interacting protein 1; RZF1, ring zinc finger 1; *CBR*, *Chlorophyll b reductase*; *CBF1*, *dehydration-responsive-element-binding* (*DREB*)* protein / C-repeat binding factor 1*

As a typical climacteric fruit, phytohormones (especially ethylene) and metabolites (such as sucrose, starch, carotenoids, and flavor-related substances) change greatly during fruit ripening (Ning, et al. 2021). Many environmental and transcription factors affecting this process also have been widely investigated and have laid an important foundation for the control of ripening among climacteric fruits (Fig. 2A).

Ethylene is crucial in regulating the ripening of banana fruits (Shan, et al. 2020b). Therefore, the inhibitor of ethylene receptor, 1-methylcyclopropene (1-MCP), is widely used to extend the shelf-life of bananas by suppressing ethylene production (Zhu, et al. 2020b). During fruit ripening, transcriptional levels of ethylene biosynthesis genes, such as *1-aminocyclopropane-1-carboxylic acid synthase* (*ACS*) and *1-aminocyclopropane-1-carboxylic acid oxidase* (*ACO*), were upregulated (Wu, et al. 2024a). The expression level of *ACO* in fast ripening banana cultivars was much higher, while the up-regulation of *ACS1* and *ACO1* genes during fruit ripening was delayed in slow-ripening cultivars (Netlak, et al. 2021). It was reported that the B genome composition of banana cultivars contributed positively to accelerating fruit ripening (Netlak, et al. 2021). Consistently, comparative analyses of A and B subgenomes also revealed that the amount of *ACO* genes in the B subgenome was larger than that in the A subgenome, and in much higher expression levels (Wang, et al. 2019b). This can explain why banana cultivars with B subgenome composition like ‘Fenjiao’ are fast-ripening. In addition to ethylene, ABA accumulation was also closely associated with the fast ripening of ‘Fenjiao’ (Hu, et al. 2017). Moreover, brassinosteroid (BR) was discovered playing a role in controlling banana fruit ripening by modulating ethylene biosynthesis (Shan, et al. 2020a).

Besides ethylene biosynthesis genes, many other ripening-related genes, such as cell wall modification-related *polygalacturonase* (*PG*), *pectin esterase* (*PE*), *pectate lyase* (*PL*), *β-D-xylosidase* (*XYL*) and *expansin* (*EXP*), starch-degradation-related *glucan, water dikinase* (*GWD*), *starch-related phosphatase Like-Sex-Four* (*LSF*), *β-Amylase* (*BAM*), *α-Amylase* (*AMY*), and sucrose biosynthesis-related *sucrose-phosphate synthase* (*SPS*), have been identified. Their upstream transcriptional regulatory pathways are mediated by different transcription factors (TFs). Two types of regulatory circuits in fruit ripening, the NAC-type circuit and the MADS-box loop, have been identified in bananas (Lü, et al. 2018). In the NAC-type circuit, NAC activates downstream ethylene biosynthesis genes (including *ACS* and *ACO*) and other ripening-related genes like *PG* and *EXP*. In the other loop, NAC and MADS-box regulate downstream ethylene-independent ripening genes. In the study conducted by Kuang et al. (2021), 25 TFs were identified as prime candidates regulating fruit ripening. MaNAC029 modulates ethylene biosynthesis and fruit quality, and undergoes MaXB3-mediated proteasomal degradation during fruit ripening (Wei, et al. 2023b). MaNAC154 can target the promoters of *MaEXP1/2*, *MaPL2*, *MaPG1/X3* and *MaXTH5/23/28* and repress their transcription (Chen, et al. 2023c). MaMADS36 directly binds to the promoter of *MaBAM9b*, increases its transcription and starch degradation (Liu, et al. 2021). MaMYB4 was identified as a negative regulator of banana fruit ripening. It suppresses the expression of ethylene biosynthetic and cell wall modifying genes (Yang, et al. 2022). MaWRKY49 positively functions in ethylene-induced fruit ripening by activating the expression of fruit softening-related genes, such as *PLs* (Liu, et al. 2023). BEL1-LIKE HOMEODOMAIN transcription factor 1 (BEL1) interacts directly with the promoters of several cell wall and starch degradation-related genes, such as *MaAMY3*, *MaXYL32*, and *MaEXP-A8*, and plays a positive role in regulating fruit ripening (Song, et al. 2023). Brassinazole resistant1/2 (MaBZR1/2), the central TF of BR signaling, can directly bind to and repress the promoters of several cell wall modifying genes like *MaEXP2*, *MaPL2* and *MaXET5*, whose transcription levels are elevated concomitant with fruit ripening (Shan, et al. 2020a). MaIAA17-like, an auxin/indole-3-acetic acid (Aux/IAA) family member, can bind to and regulate the activities of promoters of chlorophyll, starch and cell wall degradation-related genes, as well as interact with ethylene-insensitive 3-binding F-box protein (MaEBF1), further activating the expression of *MaNOL*, *MaBAM8*, *MaPL8,* and *MaSUR14* (Chen, et al. 2023b). MabHLH6 acts as a positive regulator of fruit ripening by activating the promoters of 11 starch degradation-related genes by recognizing the E-box (CANNTG) motifs present in their promoters (Xiao, et al. 2018). C2H2 zinc finger proteins were involved in fruit ripening through transcriptional control of ethylene biosynthetic genes (Han, et al. 2016). Moreover, several bZIP TFs, such as MabZIP93 (Wu, et al. 2019) and MabZIP21 (Wu, et al. 2022), have also been reported to function in regulating ripening. Additionally, multilayered transcriptional regulatory cascades controlling ethylene biosynthesis have been discovered and received excessive attention. These include the MaMADS1–MaNAC083–MaACS1/MaACOs regulatory cascade (Wei, et al. 2023d), MaXB3-MaNAC (Wei, et al. 2023a) and others.

Recently, protein phosphorylation and MAPK-related genes were frequently discovered functioning in regulation of fruit ripening. For example, MaMPK2 interacts with and phosphorylates MabZIP93, promoting MabZIP93-mediated transcriptional activation of cell wall modifying genes (Wu, et al. 2019). MaMPK14 mediates postharvest ripening by cooperating with MaMYB4 (Yang, et al. 2022). MaMPK6-3 can phosphorylate MabZIP2 and enhance MabZIP21-mediated transcriptional activation, and MabZIP21 can enhance its role in transcriptional regulation by activating the transcription of both *MaMPK6-3* and itself (Wu, et al. 2022). MaKIN10 X1/3 can interact with MaMYB13 and enhance the MaMYB13-mediated transcriptional activation via phosphorylation during fruit ripening under low-temperature condition (Li, et al. 2023a). In addition to phosphorylation, acetylation, histone methylation and sulfoxidation also play important roles in the regulation of fruit ripening. Histone deacetylase MaHDA1 can be recruited by MaERF11 and participate in repressing *MaACO1* and *expansins* during ripening (Han, et al. 2016). MaHDA6 interacts with MaNAC154 and enhances the MaNAC154-mediated transcriptional repression capacity (Chen, et al. 2023c). Moreover, the acetylation levels of histones H3 and H4 of cell wall modification-related genes, including *MaEXP1/2*, *MaPL2*, *MaPG1/X3* and *MaXTH5/23/28*, are elevated during ripening (Chen, et al. 2023c). The transient overexpression of *MaJMJ15*, a gene encoding H3K27me3 site-specific demethylase, can promote banana fruit ripening by directly targeting several key ripening-related genes (*MaNAC1/2*, *MaACS1*, *MaACO1* and *MaEXP2*) and by decreasing global H3K27me3 (Zeng, et al. 2023). The methionine sulfoxide reductase MaMsrB2 can target, and partially repair oxidized MaNAC42, and restore its DNA-binding capacity. This reductase acts as a transcriptional activator of fruit ripening under oxidative stress by directly binding to the promoters of ripening-related genes (Yan, et al. 2021).

### Banana fruit quality

The banana industry faces several key challenges affecting fruit quality, including rapid maturation (Fig. 2B), fruit ripening/softening disorders caused by low temperature storage or transportation (Fig. 2C), and green peel ripening caused by high temperature (Fig. 2D). These challenges have attracted broad attention and been widely studied.

Previous evidence has revealed that fast-ripening banana cultivars are linked to higher expression of ethylene biosynthetic and starch metabolism genes (Wang, et al. 2019b; Netlak, et al. 2021). Techniques like CRISPR/Cas9 have been used to disrupt these genes, significantly extending shelf-life. For instance, our group created *MaACO1*-disrupted Cavendish bananas by using the CRISPR/Cas9 system, the shelf-life of transgenic fruits is approximately 4 times longer than that of WT fruits (80 d vs 21 d) under natural ripening conditions, suggesting *MaACO1* is an ideal target for creating new banana germplasms with a long shelf-life of fruit by gene editing (Hu, et al. 2021a). The transient silencing of *MaC2H2-IDD*, a transcriptional activator of cell wall and starch degradation genes, results in repressed ripening of ‘Fenjiao’ banana (Song, et al. 2024). Additionally, treatments with a 2% chitosan-montmorillonite (CTS-MMT) coating (Wantat, et al. 2022) and hydrogen water (HW) (Yun, et al. 2022) have reportedly helped maintain the postharvest quality of banana fruits.

Banana fruits are sensitive to chilling injury (CI) when stored at low temperature and/or during cold chain transportation. These conditions can cause abnormal fruit softening and browning spots on banana peels. They may result from physiological dysfunction caused by membrane lipid phase changes, oxidative damage of biomacromolecules, a respiratory metabolism abnormality, etc. (Ramírez-Sánchez, et al. 2022; Li, et al. 2023; Qin, et al. 2023; Zhu, et al. 2023; Yin, et al. 2024). MaKIN10 X1/3 are involved in the response to cold stress by phosphorylating MaMYB13, which enhances the transcription of very-long-chain fatty acids (VLCFAs) and phenylpropanoids biosynthesis-related genes, including *3-ketoacyl-CoA synthase 11* (*MaKCS11*), *4-coumarate-CoA ligase 6* (*Ma4CL6*), and *peroxisomal-CoA synthetase* (*MaAAE1*) under low temperatures (Li, et al. 2023). Cold stress can significantly inhibit the transcript and protein levels of *ethylene F-box* (*EBF1*) and *abscisic acid-insensitive 5-like* (*ABI5-like*). Their ectopic and transient overexpression in ‘Fenjiao’ promotes ethylene production, starch and cell wall degradation, and decreases fruit firmness, suggesting that suppressed expression of these genes is linked to fruit softening and ripening disorders caused by cold stress (Song, et al. 2022). Similarly, cold storage can significantly downregulate expression of *MaIAA17-like*, whose transient overexpression promotes fruit ripening by inducing softening and degreening-related genes (Chen, et al. 2023b). Low temperature stress induces various epigenetic changes (Zhu, et al. 2023), such as reducing miRNA528, which targets genes encoding copper-containing proteins and induces polyphenol oxidases (PPOs), leading to peel browning (Zhu, et al. 2020a). WRKYs are also key regulators of low temperature-induced banana peel browning (Zhu, et al. 2023). Scientists have tried various methods to reduce CIs in banana fruits during low temperature transportation and storage. Some of these treatments, including hot water (Si, et al. 2024), *Opuntia ficus indica* mucilage (OFIM) edible coating (Shinga and Fawole 2023), and epibrassinolide treatment (Li, et al. 2022a), have been reported to have the potential to alleviate CIs in the harvested fruits by different mechanisms.

During banana fruit ripening, high temperatures can lead to the failure of chlorophyll degradation in peels, causing green ripening and a significant deterioration in fruit quality. Under high temperatures, MaMYB60, a positive regulator of chlorophyll catabolic genes, such as *nonyellow coloring 1* (*MaNYC1*) and *stay-green* (*MaSGRs*), will be degraded through a RING-type E3 ligase, benzoic acid hypersensitive 1 (MaBAH1)-mediated proteasomal degradation (Wei, et al. 2023c). Protein level of the chlorophyll degradation-related enzyme, MaNYC1 is significantly decreased in banana fruits ripened under high temperature through proteasomal degradation mediated by its interacting E3 ligase, MaNIP1 (Luo, et al. 2023). Another E3 ubiquitin ligase, ring zinc finger 1 (MaRZF1), also functions in green ripening by degrading MaSGR1 (Wei, et al. 2024). As green ripening caused by high temperatures greatly reduces the marketability of banana, precautions should be taken. Fortunately, cold pretreatment has been proved in effectively alleviating green ripening of banana fruits. It was rationalized that cold pretreatment can promote chlorophyll degradation by enhancing the MaCBF1 transcriptional activation ability of the *Chlorophyll b reductase* (*MaCBR*) and *MaSGR1* genes (Xiao, et al. 2023).

## Banana stress biology

### Characterization of abiotic stress resistance-related genes in bananas

**Fig. 3:**
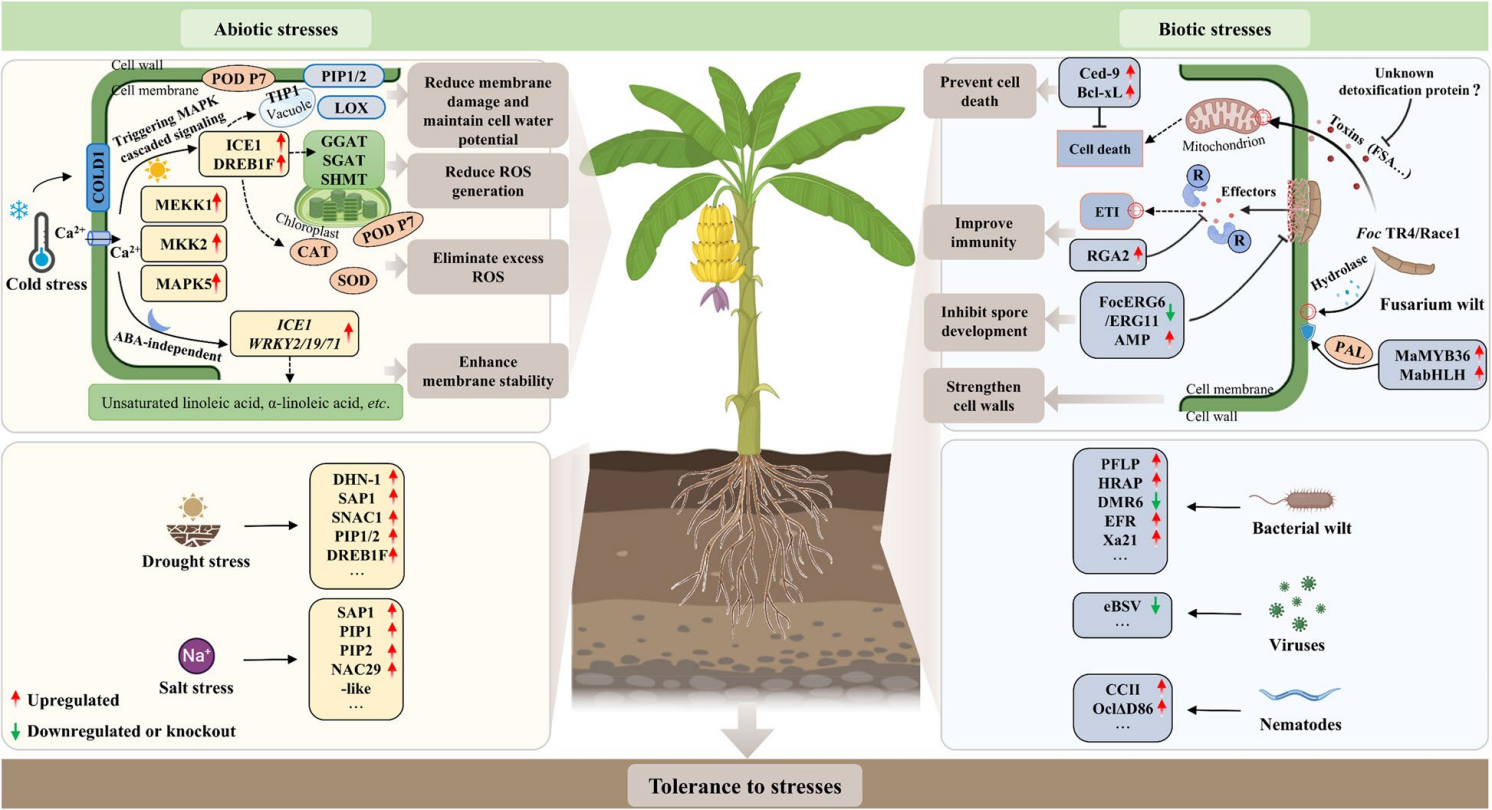
Overview of the role of key genes in diverse stressors of banana. COLD1, chilling tolerance divergence 1; MEKK1, mitogen-activated protein kinase kinase kinase 1; MKK2, mitogen-activated protein kinase kinase 2; MAPK5, mitogen-activated protein kinase 5; ICE1, inducer of CBF expression 1; DREB1F, dehydration-responsive element binding protein (DREB) transcription factor 1F; WRKY2/19/71, WRKY domain protein 2/19/71; TIP, tonoplast intrinsic protein; GGAT, glutamate glyoxylate aminotransferase; SGAT, serine glyoxylate aminotransferase; SHMT, serine hydroxymethyl transferase; LOX, lipoxygenase; CAT, catalase; SOD, superoxide dismutase; POD P7, peroxidase P7; DHN-1, dehydrin 1; SAP1, stress associated protein 1; PIP, plasma membrane intrinsic protein; PFLP, plant ferredoxin-like protein; HRAP, hypersensitive response-assisting protein; DMR6, downy mildew resistance 6; EFR, elongation factor-TU receptor; Xa21, pattern-recognition receptor; eBSV, endogenous banana streak viruses; ETI, effector-triggered immunity; RGA2, resistance gene analog 2; ERG6, *C*-24 sterol methyltransferase 6; ERG11, cytochrome P450 lanosterol *C*-14α-demethylase; AMP, antimicrobial peptide; MYB36, MYB domain protein 36; bHLH, basic helix-loop-helix; PAL, phenylalanine ammonia lyase; FSA, fusaric acid; *Foc* TR4, *Foc* tropical race 4

Banana plants are vulnerable to a variety of environmental challenges, including cold stress, drought stress, and salt stress. Recent omics advancements produced a large amount of information laying a foundation for exploring stress tolerance/resistance genes and towards breeding of tolerant/resistant bananas. Through *Agrobacterium*-mediated genetic transformation, some transgenic banana plants are successfully conferred with enhanced tolerance/resistance to single or multiple abiotic stresses. Previous studies by our group illustrated the associated molecular mechanisms underlying the differences in cold tolerance between the cold-sensitive ‘Cavendish’ banana (AAA genotype) and the cold-tolerant ‘Dajiao’ variety (ABB genotype) through multi-omics analyses and dataset integration followed by genetic transformation verification. Overall, MEKK1, MKK2, MAPK5, ICE1, MYBS3, PIP1/2 and POD52/P7 were demonstrated to play critical roles in the cold-tolerant ‘Dajiao’ (Yang, et al. 2012; Yang, et al. 2015; Dou, et al. 2016; Gao, et al. 2017; He, et al. 2018; Gao, et al. 2021; Wu, et al. 2024b) (Table 1). Under daytime conditions, ‘Dajiao’ responds to cold stress by enhancing its antioxidant capacity, primarily through the MAPK cascade signaling pathway. The main mechanisms in bananas’ response to the cold stress are involved in the following pathways and cellular reactions: (1) reduced reactive oxygen species (ROS) generation through the photorespiratory proteins glutamate glyoxylate aminotransferase (GGAT), serine glyoxylate aminotransferase (SGAT), and serine hydroxymethyl transferase (SHMT); (2) increased scavenging of excess ROS by superoxide dismutase (SOD), catalase (CAT), and peroxidase (POD 52 and P7); (3) prevention of membrane lipid peroxidation by lipoxygenase (LOX); and (4) maintaining cell water potential by aquaporins (MaPIP1;1, MaPIP1;2, MaPIP2;4, MaPIP2;6 and MaTIP1;3). Moreover, in darkness, we found the MAPK phosphorylation signaling pathway was predominantly induced in ‘Dajiao’ cells through an ABA-independent pathway. This activation led to an increased abundance of key TFs, including ICE1 and WRKY2/19/71, which enhanced cell membrane stability by elevating the levels of unsaturated linoleic acid and α-linoleic acids. These adaptations contributed to the improved acclimatization of ‘Dajiao’ to cold stress during the day and in the dark (Wu, et al. 2024b) (Fig. 3). *MusaDHN-1*, a SK3-type dehydrin gene, was identified as a multiple stress-inducible gene. Banana plants overexpressing it displayed enhanced drought and salinity stress tolerance (Shekhawat, et al. 2011). The overexpression of other multiple stress-inducible genes, such as *MusaSAP1* (*stress associated protein 1*), *MusaPIP1;2* (*plasma membrane intrinsic protein 1;2*), *MusaPIP2;6*, *MusaPIP2;7*, also displayed improved drought and/or salinity, and/or cold tolerance, through enhanced oxidative stress tolerance and/or reduced membrane injury (Sreedharan, et al. 2012, 2013, 2015; Xu, et al. 2020) (Fig. 3).

**Table 1:** Improved agricultural traits in transgenic bananas (*Musa* spp.)

Trait	Banana genotype	Transformation method	Promoter	Transformed gene	Potential molecular mechanism	Efficiency	References
*Foc* TR4 resistance	Cavendish cv. Grand Nain (AAA)	*Agrobacterium* + Banana ECS	pNos, pZmUbi	*RGA2* from a *Foc* TR4-resistant wild banana, *Ced-9* from nematode	Increasing resistance to *Foc* TR4 possibly through an R-gene-like cascade pathway (RGA2 lines), and by preventing fungus-induced cell death and maintaining organelle homeostasis (Ced9 lines)	Two transgenic lines (RGA2-3 and Ced9-21) appeared to be immune to *Foc* TR4 in an infected field trial over a 3-year period	(Dale, et al. 2017)
*Foc* TR4 resistance	Cavendish cv. Grand Nain (AAA)	*Agrobacterium* + Banana ECS	pZmUbi	*MpbHLH* from cold-tolerant Dajiao banana	Strengthen banana cell wall and/or scavenge ROS	Disease index of 2 transgenic plants was significantly lower than control after 2-week inoculation in pots under normal management	(Li, et al. [Bibr CR45][Bibr CR46])
*Foc* TR4 resistance	Cavendish cv. Grand Nain (AAA)	*Agrobacterium* + Banana ECS	pZmUbi	*Foc* TR4 *ERG6* double strand RNAs, *Foc* TR4 *ERG11* double strand RNAs	Induced *Foc ERG6* and *Foc ERG11* genes silencing in banana, inhibited fungal ergosterol synthesis and conidial germination	Only 30% of *ERG6*-RNAi or 15% of *ERG11*-RNAi plants were sensitive to *Foc* TR4, but more than 85% of WT showed apparent Fusarium wilt symptoms in a heavily infected field after 2 years	(Dou, et al. 2020)
*Foc* TR4 resistance	Furenzhi (AAcv)	*Agrobacterium* + Banana ECS	pCaMV35S	*ThChit42* from *Trichoderma harzianum*	Antifungal activities by cleaving chitin in the fungal cell wall	A transgenic line T3 showed no disease symptoms and remained healthy after 2 months inoculation in pots	(Hu, et al. 2013)
*Foc* TR4 resistance	Taijiao (AAA)	*Agrobacterium* + Particle bombardment + Banana Apical meristem	pCaMV35S	*Human lysozyme*	Antifungal activity by cleaving chitin in the fungal cell wall	Two transgenic lines H-67 and H-144 remained healthy and were able to fruit in the field	(Pei, et al. 2005)
*Foc* TR4 resistance	Pisang Nangka (AAB)	Particle bombardment + Banana Single cauliflower-like bodies	pCaMV35S	*OsTLP* from rice	Antifungal activities by alternating fungus cell membrane integrity leading to inhibition of fungal growth, spore lysis, reduction in spore number, or reduced viability of germinated spores	The average percentage of disease incidence in transgenic plants was 29.4% compared to the control at 89.1% after 4 weeks inoculation in pots	(Mahdavi, et al. 2012)
*Foc* TR4 resistance	Cavendish cv. Williams (AAA)	*Agrobacterium* + Banana ECS	pCaMV35S	*MaLYK1* from Williams banana	Resistance against *Foc* TR4 by mediating MAMP-induced ROS generation and defense gene activation	No obvious lesions observed in inoculated *MaLYK1*-OE lines compared with WT and *MaLYK1*-RNAi lines in pots	(Zhang, et al. 2019)
*Foc* Race1 resistance	Lady Finger (AAB)	*Agrobacterium* + Banana ECS	pZmUbi	*Bcl-xL*, *Ced-9*, *Bcl-2 3’* UTR	*Bcl-xL* and ⁄or *Ced-9* may prevent cell death and enhance plant resistance characteristics by contributing to the maintenance of organelle homeostasis; *Bcl-2 3’* UTR confers resistance to plant cells is unknown	The transgenic line *Bcl-2 3’* UTR-6 showed a level of *Foc* Race1 resistance similar to resistant wild-type ‘Grand Naine’ at least 3 months after inoculation in small-plant bioassays	(Paul, et al. 2011)
*Foc* Race1 resistance	Silk cv. Rasthali (AAB)	*Agrobacterium* + Banana ECS	pZmUbi	*MusaDAD1*, *MusaBAG1* and *MusaBI1* from Rasthali banana	*MusaBAG1* gene plays a far greater role in the control of PCD in banana plants compared to the other two genes studied	*MusaBAG1* overexpressing plants demonstrated the best resistance towards *Foc* Race1 infection in the three groups of transgenic plants derived from the three gene constructs after 6 weeks in greenhouse bioassays	(Ghag, et al. 2014a)
*Foc* Race1 resistance	Silk cv. Rasthali (AAB)	*Agrobacterium* + Banana ECS	pZmUbi	*PhDef1* and *PhDef2* from *Petunia*	Interact with specific lipids on the fungal membrane and subsequently permeabilize them to inhibit fungus growth	Four transgenic plants displayed a high degree of resistance to *Foc* Race1 challenge after 3 months inoculation in pots	(Ghag, et al. 2012)
*Foc* Race1 resistance	Silk cv. Rasthali (AAB)	*Agrobacterium* + Banana ECS	pCaMV35S	*Ace-AMP1* from onion	The activity of this AMP is mainly on the structural components of the cell wall attacking multiple targets	Six transgenic plants root challenged with *Foc* Race1 showed VDIs (vascular disease index) ranging from 38 to 48% compared to the control at 100%, after 6 months inoculation in pots	(Mohandas, et al. 2013)
*Foc* Race1 resistance	Silk cv. Rasthali (AAB)	*Agrobacterium* + Banana ECS	pCaMV35S, pZmUbi	*Ace-AMP1* from onion and *PFLP* from sweet pepper	Higher tolerance to oxidative stress caused by *Foc* Race1 infection	Two transgenic plants root challenged with *Foc* Race1 showed VDIs ranging from 10 to 20% compared to the control at 96%, after 6 months inoculation in pots	(Sunisha, et al. 2020a)
*Foc* Race1 resistance	Silk cv. Rasthali (AAB)	*Agrobacterium* + Banana ECS	pZmUbi	*Sm-AMP-D1* from *Stellaria media*	Antifungal activity by destabilizing the microbial membranes	Two transgenic lines, Sm-D1 and Sm-D2, were without external wilt symptoms after 6 months inoculation in pots	(Ghag, et al. 2014c)
*Foc* Race1 resistance	Silk cv. Rasthali (AAB)	*Agrobacterium* + Banana ECS	pZmUbi	*Foc* Race1 *VEL* intron hairpin RNAs, *Foc* Race1 *FTF1* intron hairpin RNAs	Induced *Foc VEL* and *Foc FTF1* genes silencing in banana, inhibited fungal growth, development and pathogenesis	Disease severity scale of 7 ihpRNA-*VEL* and 5 ihpRNA-*VEL* transgenic plants was less than 1 (1 = no symptoms) in a 6-week-long bioassay in pots	(Ghag, et al. 2014b)
*Foc* Race1 resistance	Silk cv. Rasthali (AAB)	*Agrobacterium* + Banana Single buds	pCaMV35S	*GmEg* from soybean	Antifungal activity by cleaving β-glucan, a component of mycelial cell walls	Performance of the two transgenic lines was better than the control after 4 weeks inoculation in pots	(Maziah, et al. 2007)
*Foc* Race2 resistance	Silk cv. Rasthali (AAB)	*Agrobacterium* + Banana ECS	pAtUbq3	*MSI99* (a magainin analogue gene)	Interacted with the microbial membrane, resulting in loss of essential metabolites and membrane depolarization and uncoupling of respiration in bacteria	Five plants expressing the peptide in the cytoplasm and 11 transgenic plants expressing the peptide in the intercellular spaces were resistant to *Foc* Race2 in pots	(Chakrabarti, et al. 2003)
Banana Xanthomonas wilt	Sukali Ndiizi and Nakinyika bananas	*Agrobacterium* + Banana ECS	pCaMV35S	Plant ferredoxin-like protein (*Pflp*) from sweet pepper	Disease resistance through formation of hypersensitivity response-like necrosis	All the transgenic lines except one showed absolute resistance to BXW after 60 days inoculation in pots	(Namukwaya, et al. 2012)
Banana Xanthomonas wilt	Sukali Ndiizi and Mpologoma bananas	*Agrobacterium* + Banana ECS	pCaMV35S	Hypersensitivity response-assisting protein (*Hrap*) gene from sweet pepper	Disease resistance resulting from enhanced hypersensitive cell death	Six transgenic lines showed absolute resistance to BXW after 60 days inoculation in pots	(Tripathi, et al. 2010)
Banana Xanthomonas wilt	Gonja manjaya banana	*Agrobacterium* + Banana ECS	pCaMV35S	*Pflp* and *Hrap* from sweet pepper	Stacking might provide of durable resistance to BXW	Seven transgenic lines with stacked genes showed complete resistance to BXW after 60 days inoculation in pots	(Muwonge, et al. 2016)
Banana Xanthomonas wilt	Dwarf Cavendish (AAA)	*Agrobacterium* + Banana ECS	pCaMV35S	ELONGATION FACTOR-TU RECEPTOR (*AtEFR*) gene from *Arabidopsis*	Enhanced resistance through activation of early immune outputs (e.g., ROS, defense gene expression) mediated by recognition of *Xcm* EF-Tu by EFR	Eighteen transgenic lines exhibited partial resistance (50–75%) compared to control after 60 days inoculation in pots	(Adero, et al. 2023a)
Banana Xanthomonas wilt	Sukali Ndiizi banana	*Agrobacterium* + Banana ECS	CRISPR/Cas9-mediated editing	Downy mildew resistance 6 (*DMR6*) gene from banana	Enhanced resistance through knockout of a susceptibility gene during pathogen infection	One inoculated plant of D15 was without BXW symptoms after 60 days inoculation in pots	(Tripathi, et al. 2021)
Banana Xanthomonas wilt	Gonja manjaya banana	*Agrobacterium* + Banana ECS	pZmUbi	*Xa21* pattern-recognition receptor from wild rice	*Xa21* receptor may recognize a microbial determinant that is conserved in *Xcm*	Twelve inoculated plants were without BXW symptoms after 60 days inoculation in pots	(Tripathi, et al. 2017)
Shelf life	Cavendish cv. Brazilian (AAA)	*Agrobacterium* + Banana ECS	CRISPR/Cas9-mediated editing	*MaACO1* from banana	Promotes the shelf life of banana fruit by inhibiting ethylene biosynthesis	*MaACO1*-disrupted fruit remained yellow or green 60 days postinoculation vs. WT fruit with brown spots at day 21	(Hu, et al. 2021)
Cold tolerance	Dajiao banana (ABB) and Cavendish cv. Grand Nain (AAA)	*Agrobacterium* + Banana ECS	pZmUbi	*MAPK3* (same as *MAPK5* in this review) and ICE1 from cold-tolerant Dajiao banana	MaMAPK5-MaICE1-MaPOD P7 pathway, a positive regulator of cold tolerance in banana	Cold tolerance of *MAPK3*-RNAi plants decreased at 10 ℃ for 7 days in pots in an ambient environment. Cold tolerance of *MaICE1*-overexpressing plants increased at 10 ℃ for 2 days followed by recovery for 3 days	(Gao, et al. 2021)
Cold tolerance	Cavendish cv. Brazilian (AAA)	*Agrobacterium* + Banana ECS	pZmUbi	*MYBS3* from cold-tolerant Dajiao banana	*MpMYBS3*-overexpressing lines had a higher proline content, accumulated less malondialdehyde and displayed lower levels of electrolyte leakage	Cold tolerance of *MpMYBS*-overexpressing plants increased at 10 °C for 2 days followed by recovery for 3 days	(Dou, et al. 2016)
Drought and salinity tolerance	Silk cv. Rasthali (AAB)	*Agrobacterium* + Banana ECS	pZmUbi	*DHN-1* from banana	Increased the protective antioxidative capacity under drought- and salt-stress conditions, reducing free radical-induced damage to the cellular membranes of transgenic plants	Transgenic lines responded significantly better after the initiation of drought or salt stress	(Shekhawat, et al. 2011)
Drought and salinity tolerance	Silk cv. Rasthali (AAB)	*Agrobacterium* + Banana ECS	pZmUbi	*SAP1* from banana	Improves capacity to scavenge free radicals under drought/salt stress conditions, reducing free radical-induced damage to the cellular membranes of transgenic plants	Small, uniform, in vitro transgenic shoots overexpressing *MusaSAP1* tolerated simulated drought and salt stress (after 10 days in rooting medium supplemented with 100 mM mannitol or 100 mM NaCl) better than the controls	(Sreedharan, et al. 2012)
Drought and salinity tolerance	Silk cv. Rasthali (AAB) and Cavendish cv. Grand Nain (AAA)	*Agrobacterium* + Banana ECS	pZmUbi	*NAC29-like* from banana	Increases JA and SA content, further elevating the antioxidant capacity of transgenic plants	Transgenic cv. Rasthali and cv. Grand Naine overexpressing *MusaNAC29-like* had superior tolerance of drought and salinity stress, but overexpression retarded growth and yield of the transgenic banana fruits	(Negi, et al. 2023)
Cold, drought and salinity tolerance	Silk cv. Rasthali (AAB)	*Agrobacterium* + Banana ECS	pZmUbi, pMusaDHN-1	*PIP1;2* from banana	Lower malondialdehyde levels, elevated proline and relative water content, and higher photosynthetic efficiency in transgenic lines under different abiotic stress conditions	Transgenic banana plants overexpressing *MusaPIP1;2* had better abiotic stress survival characteristics	(Sreedharan, et al. 2013)
Salinity tolerance	Silk cv. Rasthali (AAB)	*Agrobacterium* + Banana ECS	pZmUbi, pMusaDHN-1	*PIP2;6* from banana	Better photosynthetic efficiency and less membrane damage in transgenic lines under salt stress conditions	Transgenic banana plants overexpressing *MusaPIP2;6* used constitutive or inducible promoter led to higher salt tolerance	(Sreedharan, et al. 2015)
Drought, cold and salinity tolerance	Mas cv. Gongjiao (AA)	Particle bombardment + *Agrobacterium* + buds of immature banana male flower	pCaMV35S	*PIP2;7* from banana	Conferred tolerance by maintaining an osmotic balance, reducing membrane injury, and improving ABA levels	Transgenic banana plants overexpressing *MusaPIP2;7* improved tolerance to multiple stresse, including drought, cold, and salt	(Xu, et al. 2020)
Cold and drought tolerance	Mas cv. Gongjiao (AA)	Particle bombardment + *Agrobacterium* + buds of immature banana male flower	pCaMV35S	*DREB1F* from banana	Conferred tolerance by common modulation of the protectant metabolite levels of soluble sugar and proline, activating the antioxidant system, and promoting jasmonate and ethylene syntheses	Transgenic banana plants overexpressing *MaDREB1F* increased banana resistance to cold and drought stress	(Xu, et al. 2023)

TFs play important regulatory roles in plant stress responses. In bananas, some transgenic plants have been reportedly with enhanced abiotic tolerance/resistance. For example, the drought and salinity stress resistance of transgenic ‘Rasthali’ and ‘Grand Naine’ bananas overexpressing *MusaNAC29-like* was significantly improved by modulating phytohormone metabolism and inducing the expression of stress-responsive genes (Negi, et al. 2023). The overexpression of *MaDREB1F* can improve the cold and drought resistance of bananas by modulating protective metabolites and the antioxidant system, activating promoters of *MaAOC4* and *MaACO20* and therefore promoting jasmonate (JA) and ethylene biosynthesis (Xu, et al. 2023) (Fig. 3).

In addition to these genes, whose functions have been verified through stable genetic transformation in bananas, the heterologous expression of some other genes can also lead to the enhanced stress tolerance of transgenic plants. Transgenic *Arabidopsis* overexpressing a cold-, salt- and drought-inducible *MaROP5g* displayed increased salt tolerance (Miao, et al. 2018). The overexpression of drought-inducible *MaWRKY80* significantly improved the drought resistance of transgenic *Arabidopsis* plants by modulating the expression of *NCED* (*9-cis-epoxycarotenoid dioxygenase*) and ABA biosynthesis (Liu, et al. 2020b). Moreover, through genome-wide identification and expression analyses, some genes were also identified to be associated with abiotic stress tolerance in bananas (Jangale, et al. 2019; Meng, et al. 2020; Anuradha, et al. 2022). With the development and application of banana genetic transformation methods, the functions of these candidate stress-responsive genes will be further investigated.

### Characterization on banana biotic stress resistance-related genes

#### Fusarium wilt disease

Several known banana diseases are debilitating and drastically reduce yield. Fusarium wilt (FW), or Panama disease, caused by *Fusarium oxysporum* f. sp. *cubense* (*Foc*) is the most devastating one and can cause a 100% yield loss of many banana cultivars (Rocha, et al. 2022). Among the four races of *Foc*, three (races 1, 2, and 4) specifically affect bananas, and tropical race 4 is the most destructive one. As a semi-necrotrophic fungus, the infection process of *Foc* in banana roots involves four key steps: 1) toxins (such as phytotoxin fusaric acid, FSA) secretion to induce programmed cell death (PCD) by regulating the expression of genes involved in mitochondrial functions (Liu, et al. 2020a); 2) cell wall-degrading enzymes secretion to facilitate invasion of the fungus into plant cells; 3) haustorium expansion to ensure continuous nutrient absorption; and 4) effector secretion to destroy the immune system of the plant cells and achieve stable colonization. To mitigate this disease, various genetic engineering strategies at multiple levels have been employed (Table 1).

Despite no gene being reported to detoxify *F. oxysporum* toxin, overexpressing negative regulatory genes of the PCD pathway in transgenic plants has shown promise in preventing cell death due to pathogen attack (Ghag, et al. 2014a). For instance, ‘Lady Finger’ bananas overexpressing animal apoptosis-negative regulator genes such as *Bcl-xL*, *Ced-9*, and *Bcl-2* 3′ UTR demonstrated improved resistance to *Foc *Race1 (Paul, et al. 2011). Three apoptosis-related genes, *MusaDAD1*, *MusaBAG1*, and *MusaBI1*, have been identified, with *MusaBAG1* conferring the best resistance among transgenic ‘Rasthali’ bananas (Ghag, et al. 2014a). Additionally, overexpression of the *Ced9* gene has increased *Foc *Race1 resistance in both ‘Lady Finger’ and ‘Rasthali’ bananas (Paul, et al. 2011; Sunisha, et al. 2020b). These reports demonstrated that inhibiting cell death, or apoptosis, was an effective approach for increasing the *Foc* resistance of bananas (Magambo, et al. 2016) (Fig. 3).

Genomic and transcriptomic insights have revealed that MpMYB36 promoted the expression of lignin biosynthesis-related genes through its regulation of phenylalanine ammonia lyase (PAL), thereby strengthening cell walls in ‘Plantain’ bananas (Xie, et al. 2024). We also found that the overexpression of the *MpbHLH* gene (i.e. *ICE1* gene cloned from cold-tolerant ‘Dajiao’, ABB genotype) greatly upregulated the expression levels of stress-related genes and accumulations of PAL and POD enzymes, and consequently, two transgenic ‘Cavendish’ banana lines displayed superior resistance to *Foc* TR4 (Li, et al. 2022b) (Fig. 3).

The expression of antifungal proteins or small molecules in banana cells is an effective measure to prevent pathogenic fungi from obtaining nutrients and further invasion. Transgenic bananas overexpressing antifungal protein genes, such as *MSI99* (a magainin analogue gene), *Ace*-*AMP1*, *Stellaria media* defensin gene (*Sm*-*AMP*-*D1*), *Petunia* floral *defensins* (*PhDef1* and *PhDef2*), exhibited enhanced resistance to *Foc* and other pathogens (Chakrabarti, et al. 2003). Host-induced gene silencing (HIGS) of *Foc* genes has been applied in the genetic breeding of bananas to improve resistance against FW (Ghag, et al. 2014b). The HIGS of some vital fungal genes, such as *velvet* (*VEL*), *Fusarium transcription factor 1* (*FTF1*), *C-24 sterol methyltransferase 6* (*ERG6*) and *cytochrome P450 lanosterol C-14α-demethylase* (*ERG11*), can efficiently confer bananas with resistance to *Foc* in pots*,* especially the *ERG11*-RNAi lines created by our group, showed more apparent *Foc* TR4 resistance in a heavily infected field for 2 years (Dou, et al. 2020). Our research data not only provides an elite broad-spectrum resistance gene in banana breeding using HIGS technology, but also paves a theoretical foundation for developing double stranded RNA fungicide to control FW (Dou, et al. 2020) (Fig. 3). Heterologous expression of some hydrolases actively attacking fungal cell walls can also improve FW resistance. Some disease resistance-related genes, including the *Trichoderma harzianum* endochitinase gene *chit42* (Hu, et al. 2013) and soybean *endo β-1,3-glucanase* gene (*GmEg*) (Maziah, et al. 2007), have also been successfully used to create banana transgenic lines with improved *Foc* resistance. Stable overexpression of some human and animal genes has also reportedly enhanced the resistance of bananas to FW, the overexpression of human lysozyme (HL) has delayed FW symptoms in transgenic bananas (Pei, et al. 2005).

Host resistance is the most effective strategy for the management of *Foc*. Although function of the native R gene in resisting *Foc* TR4 has been investigated, banana native R gene specific to each of the three *Foc* races has not been identified yet. Two transgenic ‘Cavendish’ lines overexpressing *resistance gene analog 2* (*RGA2*), a gene isolated from a *Foc* TR4-resistant *M. acuminata* ssp. *malaccensis*, showed no disease symptoms during a three-year field experiment (Dale, et al. 2017) (Fig. 3), whether it is resistant to other *Foc races* is unknown. Chen et al. (2023a) reported a 959 kb region on chromosome 3 of the ‘DH-Pahang’ reference assembly v4 of *Foc* TR4 and *Foc* subtropical race 4 (*Foc* STR4) resistance using two segregating populations of *M. acuminata* ssp. *malaccensis*. Within this region, a gene encoding a leaf rust 10 disease-resistance locus receptor-like protein kinase-like 2.1 (*LRK10L-2.1*, *Macma4_03_g32220.1*) was an important candidate gene associated with *Foc* TR4 and *Foc* STR4 resistance (Chen, et al. 2023a). *MaLRR-RLP74* and *MaLRR-RLP75* were recommended as a good starting point to search genes responsible for *Foc* Race1 resistance (Álvarez-López, et al. 2022). Genomics studies demonstrated that more resistance genes, and greater dramatic expression changes of many resistance genes in response to pathogens were found in the B subgenome (Li, et al. 2024; Xie, et al. 2024), suggesting that the exploration of specific resistant genes in the B subgenome could provide another promising avenue for banana disease resistance breeding.

#### Bacterial and other diseases

In addition to FW, the banana industry is also severely undermined by diseases caused by other fungal pathogens, and by bacteria, viruses, and nematodes. Among them, Banana Xanthomonas wilt (also called Bacterial wilt disease, BXW) caused by *Xanthomonas campestris* pv. *musacearum* (*Xcm*) can destroy a plantation (Tripathi, et al. 2017). Several *R* genes and *AMPs* have been successfully transformed into bananas and identified with ability to control bacterial pathogens (Tripathi, et al. 2010; Namukwaya, et al. 2012; Tripathi, et al. 2014). Transgenic bananas overexpressing sweet pepper plant ferredoxin-like protein gene (*PFLP*)/hypersensitive response-assisting protein gene (*HRAP*) have been subjected to BXW resistance evaluation in Uganda field trails (Tripathi, et al. 2016). Transgenic banana ‘Gonja manjaya’ expressing stacked *HRAP* and *PFLP* genes also displayed improved resistance against BXW, but stacked transgenic lines showed no synergistic effect (Muwonge, et al. 2016). Moreover, the overexpression of the *Arabidopsis elongation factor-TU receptor* (*AtEFR*) gene in dwarf Cavendish displayed enhanced resistance against the BXW pathogen (Adero, et al. 2023a). Recently, it was reported that editing of the *downy mildew resistance 6* (*DMR6*) orthologue gene could confer transgenic banana with enhanced resistance to BXW (Tripathi, et al. 2021) (Fig. 3).

Nematodes and viruses are also important limiting factors in banana fruit production. It was recognized that fruit losses caused by nematodes can be up to 50% (Tripathi, et al. 2017). Banana bunchy top disease caused by Banana bunchy top virus (BBTV) is one of the most devastating banana diseases in the most banana cultivation areas. The transformation of a *cysteine proteinase inhibitor* and/or synthetic peptide in plantain can lead to enhanced resistance to nematodes in the field (Tripathi, et al. 2017) (Fig. 3). For virus diseases control, several approaches, such as post-transcriptional gene silencing (PTGS), RNA interference (RNAi) and virus-activated cell death, and CRISPR/Cas9 editing of virus genes, have been applied (Tripathi, et al. 2019).

## Molecular breeding of bananas

**Fig. 4:**
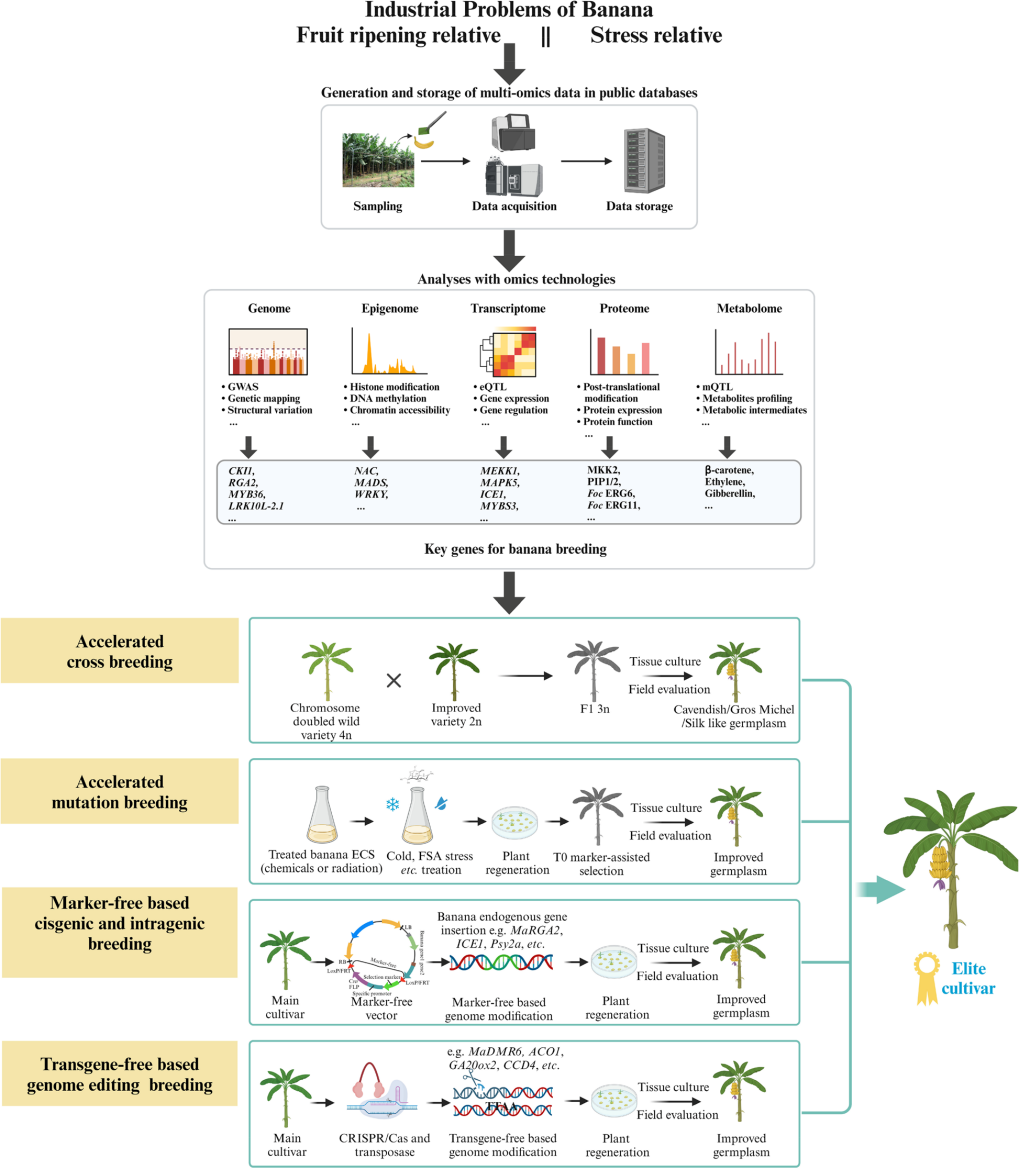
Application of multi-omics for enhancing the molecular breeding of bananas

Although most cultivated bananas are of sterile and polyploidy characteristics, banana breeding has advanced greatly in the past decades. Through adaption of somaclonal variation, artificial mutation, hybridization and ploidy breeding measures, breeders have discovered and developed many new banana cultivars. Recently, a banana marker database was released (Biswas, et al. 2024), which will greatly facilitate the identification of mutants and crossing progenies (Fig. 4).

Thanks to the continuous optimization of banana regeneration, propagation and transformation systems, plus rapid developments in genomics and high throughput sequencing techniques, the molecular breeding of bananas has accelerated rapidly. In the early years of banana genetic transformation research, apical meristems and buds were frequently used, resulting in high proportions of chimeras. Therefore, embryogenic cell suspensions (ECS) are now preferentially used as explants for banana genetic transformation (Table 1). ECSs of an increasing number of banana cultivars are successfully produced by developing and optimizing strategies for improving somatic embryogenesis (Adero, et al. 2023b).

Genetic transformation systems for bananas have been successfully established and used in the functional analysis of many fruits ripening-, quality-, and stress-related genes (as described in previous sections and Table 1). Additionally, functions of some noncoding RNAs (ncRNA) have also been validated through genetic transformation. For example, overexpression of the *Musa miR397* can enhance the plant biomass of transgenic bananas by two- to three-fold (Patel, et al. 2019). In the future, the functions of more genes and ncRNAs will be investigated using stable banana genetic transformation methods.

Although many successful cases of genetic transformation have been reported, the transformation efficiency of most banana species is still relatively low. Scientists have tried numerous methods to enhance the efficiency of various cultivars. For example, the addition of melatonin increased the transformation efficiency of ‘Grand Naine’ banana by approximately 1.5-fold (Shivani and Tiwari 2019). The application of developmental regulators (DRs) has the potential of inducing meristem and improving genetic transformation efficiency (Hao, et al. 2024). In banana, two *DR* genes, *MaBBM2* and *MaWUS2*, have been proposed as promising molecular markers of embryogenicity (Shivani, et al. 2017), indicating a potential role in improving the efficiency of banana genetic transformation.

Given its ability to create precise alterations or mutations in the plant genome, CRISPR/Cas9-based genome editing has emerged as a powerful tool for banana improvement and breeding (Tripathi, et al. 2020, 2023; Wu, et al. 2020). To increase the mutation efficiency in bananas, our group introduced an endogenous U6c promoter and a banana codon-optimized Cas9 into the CRISPR/Cas9-mediated genome editing system, resulting in a four-fold increase in mutation efficiency (Zhang, et al. 2022). To obtain marker-free or transgene-free modified bananas, steroid-inducible recombinase platform (Kleidon, et al. 2020), REG-2 promoter-driven gene-deletion system (Hu, et al. 2023), particle bombardment (Awasthi, et al. 2022) were tested and have been successfully applied in banana genome editing. CRISPR/Cas9 genome editing reveals a notable success in creating banana resources with altered agronomic traits, including the *MaGA20ox2* modified semi-dwarf (Shao, et al. 2020) and *MaACO1* disrupted shelf-life extended bananas (Hu, et al. 2021), both created by our group.

It should be noted that the transgenic ‘Pei chiao’ banana has been successfully used to produce porcine reproductive and respiratory syndrome virus (PRRSV) antigen by overexpressing the *ORF5* gene of PRRSV (Chan, et al. 2013). Pigs can be immunized with transgenic recombinant GP5 protein after being orally fed with these transgenic banana leaves. This has opened a new avenue for producing vaccines against PRRSV. Moreover, transgenic bananas are uniquely suitable for producing edible plant-based vaccines against both animal and human diseases, with importantly manufacturing and delivery advantages compared to conventional vaccines (Sharma and Sood 2011; Maji, et al. 2016).

## Challenges and future perspectives

Over the past decades, tremendous progress has been made in understanding complex genetic background and evolutionary relationship of bananas, the biology of fruit ripening, and multi-omics based stress responses, and developing powerful breeding approaches to create elite banana germplasms. For the roads ahead, cutting-edge technologies such as new multi-omics technologies, precise genome-editing tools, high-throughput specific promoter identification and transgene-free genetic transformation methods will be further developed and applied to extend our understanding of unsolved molecular biology questions and expand the genetic diversity to overcome the challenges of several biotic/abiotic stresses and fruit quality problems in banana.

### Multi-omics technologies will accelerate the progress of banana researches

Although many omics technologies have been successfully utilized to address key issues of banana, some new omics techniques have not been applied in banana yet. Bananas have a long, complex history of domestication, with more than 1,000 varieties of bananas cultivated around the world (Justine, et al. 2022). Therefore, detecting genetic differences related to their agronomic traits is very difficult. A pangenome can offer a way to explore the underlying molecular diversity and phenotypic variations in plants from the same genus and even the same family (Morgante, et al. 2007). Although a pangenome study of bananas has been reported, there are only 15 banana accessions included in the study (Rijzaani, et al. 2022). Thus, a pangenome covering more banana accessions and their relative species is especially necessary to provide a broader array of the banana origination and domestication, and therefore will be beneficial to support its breeding programs.

Current research at the single-cell level has increasingly become more common in plant science (Yu, et al. 2023). Single-cell multi-omics technologies enable to characterize cellular states and activities in organisms at the single-cell level by integrating profiles of the genome, transcriptome, proteome, metabolome, epigenome, and other sub-omes, and are revolutionizing molecular cell biology research (Baysoy, et al. 2023). These technologies enable systematic temporal comparisons for different cell types in specific tissues at various development stages and in response to various environmental and biotic stresses. They also lay a solid foundation for exploring cell-specific and tissue-specific genes and for distinguishing spatial and temporal changes of gene expression, protein and metabolite accumulations, epigenomic modifications and so on (Xia, et al. 2022; Longo, et al. 2021). We anticipate that the single-cell omics analysis will be applied for the banana research field soon.

### Overcoming the fertility barriers to promote the diversity of banana germplasm

The conventional crossbreeding of bananas for increased yield, better consumer attributes, and enhanced resistance against stressors has been hampered by inherent male and female sterility (Waniale, et al. 2024). Banana cultivars with AA and BB genotypes had higher pollen viability than banana cultivars containing triploid genomes that produce abnormal pollen (male sterility) due to partial homoeologous chromosome pairing of A and B during the prophase I of meiotic cell division. This process includes non-reducing chromosome segregates of trivalent or tetravalent pairings in anaphase I, leading to unbalanced genome transmission in gametic cells (Mingmanit, et al. 2023). In addition, non-viable pollen development in banana might be associated with the high expression of both *tapetum determinant 1* (*TPD1A*) and *MYB80* genes (Hu, et al. 2020; Mingmanit, et al. 2023). Meiotic errors, embryo sac defects, and pollen-pistil interactions contribute to female sterility (Waniale, et al. 2024). Based on a genome-wide association analysis in bananas, a putative orthologous gene to *Histidine Kinase CKI1* was identified as a strong candidate gene for female sterility (Sardos, et al. 2016). For diploid sterile bananas, the potential role of *TPD1A* and *CKI1* in male or female sterility would be of major importance for marker-assisted selection and gene modification, to cultivate fertile diploids with excellent traits. The triploid desert bananas: ‘Cavendish’ and ‘Gros Michel’, are the two most important varieties of international trade for more than one hundred years, yet their extremely low fertility has resulted in an underutilized total gene pool. If we can learn the strategy of creating clonal gamete that makes meiosis becomes mitotic (MiMe) from *Arabidopsis*, rice and tomato (d'Erfurth, et al. 2009; Wang, et al. 2019a, 2024), and so regulate the meiotic process and get clonal gametes in the triploid bananas, it may enhance their male or female fertility and produce new, heterosis, polyploid banana bred with the above modified diploid parents.

### Establishment of a transgene-free genome editing system, and exploration of new transgenic technologies

While vigorously promoting the combination of traditional banana breeding and molecular-assisted breeding methods, greater efforts should also be made to support the research and application of modern biotechnological breeding methods such as genetic engineering. Many transgenic crops have been developed using plant genetic engineering to improve insect resistance or herbicide tolerance and to enhance quality traits, but most have been used for research purposes and not yet been commercialized (Garg, et al. 2018). An *RGA2*-overexpressing Cavendish variety (QCAV-4) resistant to *Foc* TR4 was approved for commercial cultivation by the Office of the Gene Technology Regulator in Australia this year (Turrell 2024). This suggests a bright future for banana molecular breeding. To inactivate or introduce new genes during this process, especially the banana’s own genes, needs to be done in a targeted, specific manner that overcomes the technical shortcomings of traditional breeding. It must also confront the apparent negative public perception of plant genetic modification, gene editing, and *cis*-transgenesis that are gradually becoming the mainstream of banana molecular breeding, with transgene-free being the main direction of development. The main cultivated banana varieties are heterozygous triploids, and their current genetic improvement is mainly based on plant regeneration using *Agrobacterium*-mediated delivery into banana ECS cells to avoid chimera formation. Marker gene-free, trait-improved progeny have been produced using inducible or tissue-specific promoter-driven recombinase systems (Chong-Pérez, et al. 2012; Kleidon, et al. 2020; Hu, et al. 2023). Many approaches have been implemented to deliver CRISPR/Cas9 complex directly into plant cells to get transgene-free plants. For this, our group established a PEG-mediated banana protoplast transformation system based on DNA and CRISPR/Cas9 ribonucleoprotein complexes for banana (Wu, et al. 2020), but the protoplast regeneration remains a bottleneck for bananas. To date, only one study obtained transgene-free *CCD4* mutated banana by a particle bombardment method for delivery of plasmid vectors to banana ECS cells (Awasthi, et al. 2022). In our opinions, using the CRISPR/Cas9 gene-editing system in combination with a transposase (e.g. *PiggyBac*) could be a more effective way to create knockout and knock-in transgene-free bananas (Tripathi, et al. 2023; Liu, et al. 2024) (Fig. 4).

### Elite banana breeding needs tissue specific expression of important trait genes

To better manage biotic and abiotic stresses and improve the nutritional quality of banana fruits, the molecular breeding of bananas needs to express important trait genes in appropriate tissues for optimal results. Researchers developed the provitamin A-enriched golden banana using two fruit-specific promoters of the *expansin1* gene (*Exp1*) and the *ACO* gene for driving *MtPsy2a* gene expression, these biofortified transgenic bananas produce three-fold more provitamin A in the fruit peel and pulp compared to fruit from non-transgenic control plants (Paul, et al. 2017). Banana acidic chitinase class III (MaChIII) is an abundant fruit-specific storage protein that was not present in peel, corm, meristem, or root tissues, while the gene’s expression was turned off in the presence of ethylene (Suárez-Rodríguez, et al. 2022). The *MaChIII* promoter can also be used as an excellent banana fruit-specific promoter to express the target trait genes at high levels in early development of banana fruit. To ensure the specific expression of *Foc* TR4-resistant genes in banana roots. James et al. (2022) analyzed the root-specific promoters of several species in tobacco and banana, and found that the promoter of the *MaTIP2* gene was highly active in banana roots. Most cultivated bananas are vegetatively propagated triploid. It is not feasible to eliminate the integrated exogenous DNA cassette from the genome by genetic segregation following crossing or selfing. This is a limitation to the use of gene-editing techniques in molecular breeding of bananas. Our group constructed a CRISPR vector including FLP recombinase gene controlled by the embryo-specific promoter *REG-2*, can delete integrated selection marker genes without extra treatment and the regenerated bananas are marker gene-free (Hu, et al. 2023). Until now, most of the candidate tissue-specific banana promoters were strongly expressed in the target tissue, but few were truly tissue-specific (Paul, et al. 2017; James, et al. 2022). Therefore, we believe that further transcriptomic and genetic transformation experiments are highly needed to mine excellent tissue-specific promoters in the banana genome. In particular, the identification of endogenous root-specific promoters in banana combined with endogenous disease resistance genes would be very beneficial in solving the international problem of Fusarium wilt of banana through marker-free/transgene-free based genome modification (Fig. 4).

The recent advances in banana molecular biology offer means for gaining more insights in the genetics of the crops and to identifying key genes that could lead to accelerating *Musa* betterment. Sustainable breeding of new banana varieties with high-yield, high-quality, and resistance is of great importance for ensuring food security and sustainable agriculture. Further integration of these biological knowledge and exploration of new biotechnological approaches will dramatically accelerate the process of molecular breeding to produce more new elite banana varieties.

## Data Availability

All data discussed in this review are associated with the supporting primary research papers.

## Abbreviations

1-MCP: 1-Methylcyclopropene
4CL6: 4-Coumarate-CoA ligase 6
AAE: Peroxisomal-CoA synthetase
ABA: Abscisic acid
ABI5-like: Abscisic acid-insensitive 5-like
ACO: 1-Aminocyclopropane-1-carboxylic acid oxidase
ACS: 1-Aminocyclopropane-1-carboxylic acid synthase
AMPs: Antimicrobial peptides
AMY: α-Amylase
Aux/IAA: Auxin/Indole-3-acetic acid
BAG: Bcl-2-associated athanogene
BAH: Benzoic acid hypersensitive
BAM: β-Amylase
BBTV: Banana bunchy top virus
BI1: Bcl-2-associated X protein (BAX) inhibitor
BR: Brassinosteroid
BXW: Banana Xanthomonas wilt
BZR: Brassinazole resistant
CAT: Catalase
CBR: Chlorophyll *b* reductase
CCD: Carotenoid cleavage dioxygenase
ChIII: Acidic chitinase class III
CI: Chilling injury
CKI: Histidine kinase
COR: Cold regulated gene
CTR: Constitutive triple response
CTS-MMT: Chitosan-montmorillonite
DAD: Defender against death domain
DH: Doubled-haploid
DHN: Dehydrin gene
DMR: Downy mildew resistance
DRs: Developmental regulators
EAHB: East African highland banana
EBF: Ethylene-insensitive-binding F-box protein
ECS: Embryogenic cell suspension
EFR: Elongation factor-TU receptor
EIL: Ethylene insensitive-like
ERG6: *C*-24 sterol methyltransferase 6
ERG11: Cytochrome P450 lanosterol *C*-14α-demethylase
ERS: Ethylene response sensor
EXP: Expansin
*Foc*: *Fusarium oxysporum* f. sp. *cubense*
*Foc *TR4: *Foc* tropical race 4
*Foc ** S*TR4: *Foc* subtropical race 4
FSA: Fusaric acid
FTF: Fusarium transcription factor
FW: Fusarium wilt
GGAT: Glutamate glyoxylate aminotransferase
GmEg: Soybean endo β-1,3-glucanase
GWD: Glucan, water dikinase
HAD: Histone deacetylase
HIGS: Host-induced gene silencing
HL: Human lysozyme
HRAP: Hypersensitive response-assisting protein
HW: Hydrogen water
ICE1: Inducer of CBF expression 1
KCS: 3-Ketoacyl-CoA synthase
LOX: Lipoxygenase
LRK10L: Leaf rust 10 disease-resistance locus receptor-like protein kinase-like
LSF: Starch-related phosphatase Like-Sex-Four
MAN: Mannanase
MAPK: Mitogen-activated protein kinase
MEKK: MEK kinase
MiMe: Mitosis instead of meiosis
MKK: Mitogen-activated protein kinase kinase
NCED: 9-Cis-epoxycarotenoid dioxygenase
NYC: Nonyellow coloring
OFIM: *Opuntiaficusindica* Mucilage
PAL: Phenylalanine ammonia lyase
PCD: Programmed cell death
PE: Pectin esterase
PFLP: Plant ferredoxin-like protein
PG: Polygalacturonase
PhDef: *Petunia* floral defensing
PIP: Plasma membrane intrinsic protein
PL: Pectate lyase
POD: Peroxidase
PRRSV: Produce porcine reproductive and respiratory syndrome virus
PTGS: Post-transcriptional gene silencing
RGA2: Resistance gene analog 2
RNAi: RNA interference
ROP: Rho-like GTPase
ROS: Reactive oxygen species
RZF: Ring zinc finger
SAP: Stress associated protein
SGAT: Serine glyoxylate aminotransferase
SGR: Stay-green
SHMT: Serine hydroxymethyl transferase
SNP: Single nucleotide polymorphism
SOD: Superoxide dismutase
SPS: Sucrose-phosphate synthase
TFs: Transcription factors
Thchit: *Trichoderma harzianum* endochitinase
TPD1: Tapetum determinant 1
VEL: Velvet
VLCFAs: Very-long-chain fatty acids
*Xcm*: *Xanthomonas campestris* pv. *musacearum*
XET: Xyloglucan endotransglycosylase
XTH: Xyloglucan endotransglucosylase/hydrolase
XYL: β-D-xylosidase
ncRNA: Noncoding RNA
